# Multivariable Analysis of Factors Affecting Length of Stay and Hospital Charges After Atlantoaxial Fusion

**DOI:** 10.7759/cureus.1173

**Published:** 2017-04-18

**Authors:** Jian Guan, Michael Karsy, Meic Schmidt, Andrew T Dailey, Erica Bisson

**Affiliations:** 1 Department of Neurosurgery, University of Utah

**Keywords:** atlantoaxial fusion, multivariable analysis, length of stay, hospital charges, comorbidities

## Abstract

**Background:**

Atlantoaxial fusion is an effective procedure for treating degenerative, traumatic, and congenital abnormalities that result in upper cervical instability; however, data on which factors affect the length of stay and hospitalization-related charges are limited. The purpose of this study was to evaluate the pre-, intra-, and postoperative variables that affect these healthcare cost factors for patients undergoing posterior atlantoaxial fusion.

**Methods:**

We retrospectively identified from a clinical database 59 patients who underwent isolated posterior atlantoaxial fusion at a single institution from 2010 to 2015. Demographic, clinical, and surgical variables from a clinical database were analyzed with respect to the length of hospital stay and hospital charges. T-test and Chi-square testing, as well as univariate and multivariable analysis, were performed with p<0.05 considered significant.

**Results:**

On multivariable analysis, a variety of preoperative, intraoperative, and postoperative variables were associated with prolonged hospitalization and higher hospital charges, including tobacco use, preoperative diagnosis, operating room time, and the need for intraoperative blood transfusion.

**Conclusions:**

Varied preoperative, intraoperative, and postoperative factors are associated with increased length of hospitalization and higher hospital charges after atlantoaxial fusion. Familiarity with these factors may allow for improved surgical planning and patient counseling.

## Introduction

Because of its complex anatomy, the atlantoaxial region is uniquely vulnerable to certain degenerative, traumatic, and neoplastic pathological conditions [1]. Posterior cervical fusion is one of the multiple surgical options that have allowed for a significant reduction in the need for external bracing using “halo” systems [2]. Although fusion rates after posterior cervical fusion range from 93% to 99% [3] and rates of complications are low (2%–4%) [4], there is a lack of information regarding what factors influence the length of hospitalization itself [5]. An understanding of such influences is becoming increasingly important as surgical care becomes more outcomes focused [6], and measures, such as length of stay (LoS), are associated with the incidence of more serious complications, such as postoperative infection [7] and thrombotic events [8]. In addition, LoS is thought to be a major driver of health care costs, a growing cause of concern among healthcare providers and policy makers alike [9]. As value-based care gains more traction, careful attention to hospital charges and what can be done to minimize them, especially those associated with expensive surgical interventions, becomes increasingly valuable [10]. In this study, we examined various pre-, intra-, and postoperative factors that may be associated with LoS and hospital charges following posterior atlantoaxial fusion.

## Materials and methods

### Study population

After obtaining approval from the University of Utah Institutional Review Board with a waiver of informed consent, we queried a clinical database to obtain information about patients 18 years and older who underwent C1-C2 posterior spinal fusion between May 1, 2010, and April 30, 2015. This information was then confirmed and supplemented by individual chart review. Patients were excluded if other procedures (subaxial fusion, laminectomy, etc.) were performed during the same procedure or hospitalization. All surgical procedures were performed by one of five experienced spine surgeons.

### Data collection

Demographic, intraoperative, and postoperative information were collected on all patients. Demographic data included age, sex, body mass index (BMI), American Society of Anesthesiologists (ASA) Physical Status Classification System Grade, active tobacco use, active alcohol use, preoperative opioid use, employment status, marriage status, and insurance type. Relevant medical history including the presence of rheumatoid arthritis, Type 2 diabetes, hypertension, pulmonary comorbidities (i.e., asthma, chronic obstructive pulmonary disease, pulmonary embolism, bronchitis), cardiac comorbidities (i.e., atrial fibrillation, heart murmur, arrhythmia, myocardial infarction, coronary artery disease, congestive heart failure, mitral valve prolapse), previous cervical spine surgeries, and the presence of polytrauma was also obtained. Preoperative diagnoses were recorded as trauma (e.g., Jefferson fractures, odontoid fractures), instability (due to rheumatoid arthritis or congenital causes), or degenerative disease (e.g., symptomatic facet arthropathy).

Intraoperative variables included surgical time, estimated blood loss, intraoperative blood transfusion, intraoperative crystalloid and colloid infusion volumes, intraoperative complications, and axial instrumentation type. Postoperative variables consisted of the need for readmission within 30 days, postoperative complications, and discharge destination. Discharge destinations included home, skilled nursing facility, acute rehabilitation facility, and psychiatric facility, which were subsequently dichotomized to those discharged to home and to other destinations. Finally, charge data for each hospitalization was obtained.

### Statistical analysis

Continuous variables in all cases were analyzed utilizing Student’s t-test, while categorical variables were analyzed utilizing Chi-squared analysis. We performed both univariate and multivariable analysis for LoS and hospital charges. For univariate analysis of LoS, we dichotomized hospitalization length into “extended” (> 4 days) and “normal” (≤ 4 days) based on hospitalizations longer than the 75th percentile in keeping with previous studies [11]. For multivariable analysis, LoS was examined as a continuous variable. We utilized a stepwise regression analysis to construct our model, with a threshold p < 0.20 for inclusion and a p < 0.05 being defined as statistically significant in our final model. For univariate analysis of hospital charges, we dichotomized charges into “high” (> $62,200) and “normal” (≤ $62,200) based on charges greater than the 75th percentile. For multivariable analysis, hospital charges were examined as a continuous variable. We utilized a stepwise regression analysis to construct our model, with a threshold p < 0.20 for inclusion and a p < 0.05 being defined as statistically significant in our final model.

All statistical analysis was performed using SPSS V20.0 (IBM Corp., Armonk, NY). The Strengthening the Reporting of Observational Studies in Epidemiology (STROBE) guidelines were used during the preparation of this work [12].

## Results

### Length of stay

Among the 59 patients who met our inclusion and exclusion criteria, mean LoS was 4.2 days (95% confidence interval of 2.94 to 5.5 days). For our univariate analysis, 13 patients met the criterion for “extended” LoS with an average LoS of 9.2 days and 46 met the criterion for “normal” LoS with an average LoS of 2.8 days. Demographic factors were largely similar between the normal and extended groups (Table 1), including age, BMI, sex, marital status, insurance status, and employment status. ASA grade was significantly higher in the extended stay group (p = 0.026); patients in the normal LoS group were less likely to be on opioids preoperatively (15% vs 48%, p = 0.036), although other comorbidities were not statistically different between normal and extended LoS patients. There was no significant difference between the two groups in preoperative surgical diagnosis. The rate of polytrauma did not differ between the groups (13% extended LoS vs. 6% normal LoS, p = 0.602).

**Table 1:** Univariate Analysis for Extended Versus Normal Length of Stay Groups SD: standard deviation; BMI: body mass index; ASA: American Society of Anesthesiologists *P value < 0.05 was considered significant.

		Extended (N=13)	Normal (N=46)	p value*	
No. of female patients (%)		6 (46)	25 (54)	0.601	
Mean age in years ± SD		63 ± 19.1	59 ± 18.5	0.446	
BMI ± SD		27 ± 9.4	27 ± 6.0	0.851	
Married (%)		9 (69)	29 (63)	0.681	
Private insurance (%)		4 (31)	20 (44)	0.410	
Employment status (%)	Unemployed	3 (23)	15 (33)	0.768	
	Employed	3 (23)	12 (26)		
	Retired	7 (54)	18 (39)		
	Student	0 (0)	1 (2)		
ASA category (%)	Low (1-2)	4 (31)	30 (65)	0.026	
	High (3-5)	9 (69)	16 (35)		
Tobacco use (%)		2 (15)	3 (7)	0.311	
Alcohol use (%)		7 (54)	16 (35)	0.213	
Preoperative opioid use (%)		2 (15)	22 (48)	0.036	
Diagnostic category (%)	Trauma	8 (62)	16 (35)	0.216	
	Degenerative	2 (15)	14 (23)		
	Instability	3 (23)	16 (35)		
Rheumatoid arthritis (%)		0 (0)	9 (20)	0.083	
Type 2 diabetes (%)		5 (39)	8 (17)	0.106	
Hypertension (%)		3 (23)	11 (24)	0.950	
Pulmonary comorbidity (%)		4 (31)	13 (28)	0.860	
Cardiac comorbidity (%)		4 (31)	13 (28)	0.860	
Previous cervical spine surgery (%)		2 (15)	10 (22)	0.615	
Polytrauma (%)		1 (13)	1 (6)	0.602	
Estimated blood loss (ml) ± SD		263.9 ± 182.8	243.6 ± 266.0	0.798	
Intraoperative crystalloid volume (ml) ± SD		1931 ± 773.9	2415 ± 943.0	0.096	
Intraoperative colloid volume (ml) ± SD		38.5 ± 138.7	76.1 ± 234.9	0.585	
Room time (minutes) ± SD		280.1 ± 82.6	328.5 ± 108.9	0.143	
Need for intraoperative blood transfusion (%)		1 (8)	1 (2)	0.332	
Intraoperative complications (%)		3 (23)	0 (0)	0.001	
Axial screw type	Laminar	0 (0)	7 (15)	0.056	
	Pedicle	2 (15)	3 (7)		
	Pars	10 (77)	19 (41)		
	Transarticular	1 (8)	4 (9)		
	Combination	0 (0)	13 (28)		
Postoperative complications (%)		3 (23)	2 (4)	0.032	
30-day readmission (%)		1 (8)	1 (2)	0.332	
Hospital charges ± SD		$97,867.23 ± 87,665.70	$49,825.51 ± 18.795.21	0.001	
Discharge destination	Home	6 (46)	37 (80)	0.014	
	Other	7 (54)	9 (20)		

Intraoperatively, patients in the extended stay group were more likely to have experienced complications (23% vs. 0%, p = 0.001). A total of three intraoperative complications were noted—two dural tears and one vertebral artery injury—resulting in an overall intraoperative complication rate of 5.1%. Other intraoperative factors, including estimated blood loss, volume of crystalloid or colloid infused, operative room time, need for blood transfusion, and axial screw type, were not significantly different between the two groups, although there was a trend towards laminar screws in the normal LoS group and pars screws in the extended LoS group (p = 0.056).

Postoperatively, patients in the normal LoS group were significantly more likely to be discharged home than those in the extended LoS group (p = 0.001). There were five postoperative complications—one wound infection requiring washout, one patient with anemia requiring blood transfusion, one patient who developed supraventricular tachycardia, one case of intravenous infiltration requiring operative debridement, and one aspiration event requiring reintubation—resulting in an overall rate of 8.5%. Patients in the extended LoS group were significantly more likely to suffer from postoperative complications than the normal LoS group (23% vs 4%, p = 0.032).

Utilizing stepwise regression to identify predictive factors for our multivariable model, we found polytrauma, discharge to a location other than home, tobacco use, and the need for intraoperative blood transfusion were associated with a significantly longer length of stay, while higher intraoperative crystalloid volumes and the presence of pulmonary comorbidities were associated with a significantly shorter length of stay (Table 2).

**Table 2:** Multivariable Analysis of Factors Influencing Length of Stay *P value < 0.05 was considered significant

Variable	β coefficient	t statistic	p value*
Discharge destination	3.807	4.036	< 0.001
Polytrauma	13.052	5.500	< 0.001
Tobacco use	6.160	4.002	< 0.001
Crystalloid volume	-0.001	-2.290	0.026
Intraoperative blood transfusion	5.946	2.624	0.011
Pulmonary comorbidity	-2.040	-2.170	0.035

Average charges for the extended LoS group were significantly higher than that for the normal LoS group ($109,878.15, 95% CI ± $46,235.78 vs. $49,825.51, 95% CI ± $2,505.94, p < 0.001). Hospital charge data were not included in our multivariable analysis of LoS as we believe it unlikely that charges, tallied after discharge, could have any causal effect with prolonged hospitalization.

### Hospital charges

The mean hospital charge for our patient cohort was $60,410.98 (95% confidence interval of $48,002 to $72,820). For our univariate analysis, 14 patients met the criterion for “high” hospital charges with an average value of $109,878.15, and 45 met the criterion for “normal” hospital charges with an average value of $45,021.19. Demographic factors were largely similar between the high and normal hospital charge groups (Table 3), although BMI was significantly lower in the high-charge group (23.5 ± 4.2 vs. 27.8 ± 7.1, p = 0.035). The rates of most major comorbidities were similar in the high- and normal-charge groups, but the high-charge group had a higher rate of tobacco use (21% vs 4%, p = 0.046) and cardiac comorbidities (57% vs 20%, p = 0.007). Patients in the high-charge group were also much more likely to have surgery for a traumatic cause rather than instability or degenerative disease (86% vs 27%).

**Table 3:** Univariate Analysis for High Versus Normal Hospital Charge Groups SD: standard deviation; BMI: body mass index; ASA: American Society of Anesthesiologists *P value < 0.05 was considered significant.

		High (N = 14)	Normal (N = 45)	p value*
No. of female patients (%)		6 (43)	25 (56)	0.406
Mean age in years ± SD		56 ± 23.9	61 ± 16.7	0.395
BMI ± SD		23 ± 4.2	28 ± 7.1	0.035
Married (%)		7 (50)	32 (71)	0.145
Private insurance (%)		5 (36)	19 (42)	0.665
Employment status (%)	Unemployed	5 (36)	13 (29)	0.298
	Employed	3 (21)	12 (27)	
	Retired	5 (36)	20 (44)	
	Student	1 (7)	0 (0)	
Asa category (%)	Low (1-2)	5 (36)	29 (64)	0.058
	High (3-5)	9 (64)	16 (36)	
Tobacco use (%)		3 (21)	2 (4)	0.046
Alcohol use (%)		3 (21)	20 (44)	0.123
Preoperative opioid use (%)		5 (36)	19 (42)	0.665
Diagnostic category (%)	Trauma	12 (86)	12 (27)	< 0.001
	Degenerative	1 (7)	15 (33)	
	Instability	1 (7)	18 (40)	
Rheumatoid arthritis (%)		0 (0)	9 (20)	0.069
Type 2 diabetes (%)		4 (29)	9 (20)	0.499
Hypertension (%)		4 (29)	10 (22)	0.626
Pulmonary comorbidity (%)		6 (43)	11 (24)	0.184
Cardiac comorbidity (%)		8 (57)	9 (20)	0.007
Previous cervical spine surgery (%)		4 (29)	8 (18)	0.381
Polytrauma (%)		1 (7)	1 (2)	0.374
Estimated blood loss (mL) ± SD		335.6 ± 138.3	312.3 ± 93.5	0.472
Intraoperative crystalloid volume (mL) ± SD		2592.9 ± 1105.6	2220 ± 855.6	0.190
Intraoperative colloid volume (mL) ± SD		107.1 ± 289.5	55.6 ± 191.4	0.442
Room time (minutes) ± SD		312.3 ± 93.5	335.6 ± 138.3	0.472
Need for intraoperative blood transfusion (%)		2 (14)	0 (0)	0.010
Intraoperative complications (%)		1 (7)	2 (4)	0.688
Axial screw type	Laminar	1 (7)	6 (13)	0.257
	Pedicle	0 (0)	5 (11)	
	Pars	10 (71)	19 (42)	
	Transarticular	0 (0)	5 (11)	
	Combination	3 (21)	10 (22)	
Postoperative complications (%)		3 (21)	2 (4)	0.046
30-day readmission (%)		1 (7)	1 (2)	0.374
Discharge destination	Home	6 (43)	37 (82)	0.004
	Other	8 (57)	8 (18)	
Length of stay	Normal	7 (50)	39 (87)	0.004
	Extended	7 (50)	6 (13)	

Intraoperatively, patients in the high-charge group were more likely to have had a blood transfusion (14% vs 0%, p = 0.01). Other intraoperative factors were similar between the two groups. Postoperatively, patients in the high-charge group were significantly more likely to be discharged to a location other than home (p = 0.004) and to experience postoperative complications (p = 0.046). Utilizing the same dichotomization scheme as described above, patients in the high-charge group were also significantly more likely to fall into the extended LoS category (p = 0.004)

Utilizing stepwise regression to identify predictive factors for our multivariable model, we found prolonged LoS, a preoperative diagnosis of trauma, longer operative time, and postoperative complications were associated with a significantly higher hospital charges, while patients who required readmission within 30 days had significantly lower initial hospital charges (Table 4).

**Table 4:** Multivariable Analysis of Factors Influencing Hospital Charges *P value < 0.05 was considered significant

Variable	β coefficient	t statistic	p value*
Length of stay	8647.35	15.935	< 0.001
Diagnostic category	6678.21	2.154	0.036
Operative time	77.50	3.105	0.003
30-day readmission	-63962.10	-3.515	0.001
Postoperative complication	31934.18	2.705	0.009

## Discussion

The length of stay is a critical care measure after spine surgery for several reasons, ranging from its association with postoperative complications to increases in costs. Patients frequently ask how long they will be hospitalized after their operation, but this question is often difficult to answer, and evidence suggests that different factors play a role in determining the length of stay depending on what surgery is performed [5, 13]. From an economic point of view, patients in our extended stay group accrued an average of nearly $50,000 more in hospital charges compared with those in the normal LoS group. With renewed focus on value in healthcare, determination of what factors may influence LoS becomes critical. For similar reasons, hospital charge drivers will likely become a steadily increasing area of interest in the next several years, with high-cost surgical interventions being the most heavily scrutinized.

In our study, preoperative factors associated with longer LoS on multivariable analysis included tobacco use and the presence of polytrauma. Tobacco use is associated with a wide array of surgical morbidities and mortality [14-16], including increased rates of intubation, infection, and myocardial infarction [17]. All of these complications frequently result in the need for extended hospitalization, and therefore, this relationship is unsurprising. This association, combined with evidence that preoperative [16] and even postoperative [18] smoking cessation can have benefits in patients undergoing spinal procedures, should prompt aggressive patient education on the hazards of tobacco use in relation to surgery. Patients experiencing polytrauma often require hospitalization for a variety of reasons and may undergo multiple surgical interventions. The extent of injury in these patients [19] combined with frequently higher rehabilitation needs [20] likely combine to necessitate longer hospitalizations.

Interestingly, the only preoperative variable associated with a shorter length of stay was the presence of a pulmonary comorbidity. Although preoperative pulmonary dysfunction is frequently a concern in patients, it is possible that, because of its distance from the diaphragm, cervical procedures such as atlantoaxial fusion may be uniquely less susceptible to the impact of such comorbidities [21]. This fact, combined with a higher degree of vigilance in these patients in relation to postoperative mobilization and incentive spirometry use, may combine to result in a paradoxical reduction in pulmonary-related complications and thus decreased LoS.

Intraoperatively, the need for blood transfusion and lower crystalloid infusion volumes were associated with longer LoS. Evidence suggests that under-resuscitation may lead to higher rates of complications following surgical intervention [22-23]. It is possible that patients who lose enough blood intraoperatively to require transfusion and those who receive less crystalloid are at higher risk for falling into this “under-resuscitated” category. This finding suggests that surgeons and anesthesiologists should be vigilant during surgery of the possibility of under-resuscitation and correct any evidence of significant hypovolemia or anemia aggressively.

Postoperatively, inability to discharge home was associated with a significantly longer length of hospitalization. This is in keeping with previous reports of factors that are associated with longer LoS [5]. There are several possible explanations for this relationship. One is that patients who are unable to be discharged home are simply more ill and therefore require a longer time to recover following surgery. Another is that the time it takes to arrange discharge to a facility or rehabilitation unit may itself contribute to prolonged hospitalization. Regardless, careful disposition planning in these patients preoperatively—including discussions with the patient and the patient’s caregivers—is likely prudent. Postoperative complications were also associated with increased length of stay. While this could be explained simply by the need for hospitalization to manage complications that arise (the aspiration event requiring reintubation), it is also possible that a longer hospital stay may itself lead to complications. For instance, had the patient with intravenous infiltration been discharged earlier and the intravenous line removed, this incident would have been avoided. This further highlights the benefits of timely discharge to avoid possible events related to the hospitalization itself.

In relation to charges, patients with a preoperative diagnosis of trauma were more likely to incur higher charges during their hospital stay. This is possibly related to the higher likelihood of an unplanned/ emergent surgery in these patients and the inherent costs therein [24]. Patients with cardiac comorbidities and those who were active tobacco users were also more likely to fall into the higher charge group. One possible explanation for this is an increased severity of illness allowing for a higher complication/comorbidity tier to be billed for the hospitalization. Unfortunately, our charge data are not granular enough to definitively show such differences, although this would be an interesting avenue for further study. Intraoperatively, longer operative times were associated with higher hospital charges, likely related to the direct costs of time in the operating room itself.

Postoperatively, LoS and postoperative complications were both associated with increased hospital charges. Charges for room and board, nursing, medications, and other fees are the likeliest explanation of the relationship between LoS and hospital charges. The higher charges associated with postoperative complications likely result from the resources needed to address these complications—return to the operating room, subspecialist consultations, etc. Surprisingly, patients who required readmission within 30 days had significantly lower hospital charges compared with those who did not. One possible explanation for this is that these patients returned home earlier, thus incurring lower charges, but were subsequently readmitted because of issues related to such rapid discharge.

Modern techniques for atlantoaxial fixation have shown dramatic improvements in rates of fusion compared with the early, wiring-based approaches in both adults [25] and children [26]. Complication rates for these procedures are also low [27], although intimate knowledge of the local anatomy is paramount [28-29]. Despite these technical advances, data on patient and medical utilization–related outcomes remain scant. With healthcare-related costs coming into sharper focus in the past several years, the need to better define what factors influence cost drivers, such as duration of hospitalization and overall hospital charges, is becoming increasingly critical. We report one of the largest series of C1-C2 fusions at a single institution in the literature to date and the largest examining LoS and hospital charges as they relate to preoperative, intraoperative, and postoperative factors.

Our study has several limitations. The study is retrospective, and the sample size, while large for investigations of atlantoaxial fusion in the current literature, remains fairly limited. It is possible that some of the variables that were significantly associated with LoS on univariate analysis would have been found to be significant on multivariable analysis with a larger cohort, although this also suggests that the effect size would also be smaller. We hope that our findings help to guide further inquiries into factors that may be associated with higher hospital charges and increased LoS in this population, preferably utilizing data from multiple centers and significantly larger patient cohorts. Our study was limited to patients who received only isolated atlantoaxial fusions; although this likely reduced the influence of confounding factors, it also reduces generalizability to patients who require additional interventions.

Finally, our study examined charge data, a measure that, despite having some utility as a proxy for care costs [30], is not a substitute for actual cost data. The utility of charge data also varies based on the nature of payment for care, with a greater relevance in environments where “per-diem” and cash-for-service models are common versus those in which diagnosis-related group reimbursement is the norm. This fact also reduces the generalizability of our findings.

## Conclusions

Posterior atlantoaxial fusion is a well-tolerated procedure with a low rate of complications. Various preoperative, intraoperative, and postoperative factors play a role in determining which patients will require a prolonged hospitalization and accrue higher hospital charges during their stay. Familiarity with these variables may help guide preoperative planning and patient counseling in those undergoing atlantoaxial fusion.
